# Effect of anthelmintic treatment strategy on strongylid nematode species composition in grazing lambs in Scotland

**DOI:** 10.1186/s13071-016-1493-6

**Published:** 2016-04-11

**Authors:** Lynsey A Melville, David McBean, Alex Fyfe, Sara-Jane Campbell, Javier Palarea-Albaladejo, Fiona Kenyon

**Affiliations:** Moredun Research Institute, Pentlands Science Park, Bush Loan, Penicuik, EH26 0PZ Scotland UK; Biomathematics and Statistics Scotland, The King’s Buildings, Peter Guthrie Tait Road, Edinburgh, EH9 3FD Scotland UK

**Keywords:** Gastrointestinal nematodes, Lambs, Species prevalence, Anthelmintic, Multiplex PCR

## Abstract

**Background:**

Refugia based anthelmintic protocols aim to reduce the rate of development of anthelmintic resistance in gastrointestinal nematodes (GIN). Previous studies have illustrated the impact of different drenching regimes on drug efficacy and animal growth; however, the impact on nematode populations has yet to be characterised within natural infections. This study investigated the changes in species composition of GIN throughout the grazing season, following implementation of four different ivermectin drenching regimes over six years: neo-suppressive monthly treatment (NST), targeted selective treatment (TST), strategic prophylactic treatment (SPT) and treatment upon observation of clinical signs (MT).

**Methods:**

Lambs were grazed on one of eight replicate paddocks each grazing season following treatment regimes assigned in year 1. Faecal samples were collected fortnightly from all animals and hatched to first stage larvae (L_1_). DNA was extracted from individual L_1_ and a multiplex PCR assay targeting the internal transcribed spacer 2 (ITS2) region of *Teladorsagia circumcincta*, *Trichostrongylus* spp. and *Haemonchus contortus* conducted*.* Other species were identified using species-specific PCR. Worm-naïve tracer lambs were grazed on the paddocks at the start and end of each grazing season and adult worms recovered at post mortem to investigate the parasite population on pasture.

**Results:**

Results showed an overall decrease in species diversity in egg output from the NST group which occurred within a single grazing season and was consistent throughout the experiment. Species diversity was protected over six years in groups implementing TST, SPT and MT treatment regimes, designed to offer refugia. The expected shift in species prevalence throughout the season from *Teladorsagia* to *Trichostrongylus* was observed in all but the NST group where only *Teladorsagia* spp. were recovered from trial lambs by the end of the experiment. Worm burdens indicated the presence of several species at relatively low abundance on pasture in the NST group in 2011. However, these species were not represented in egg output from trial lambs, probably due to the frequent anthelmintic treatment administered throughout the grazing season.

**Conclusion:**

The molecular methods utilised here worked well. The comparable results of the three refugia-based treatment regimes suggest that nematode diversity can be maintained using part or whole group treatments if a rich supra-population of parasites are available to re-infect animals post treatment.

## Background

Gastrointestinal nematodes (GIN) represent some of the most economically important infections of livestock worldwide. GIN encompasses a wide range of parasitic worms found within the digestive tract of their host; symptoms include acute diarrhoea, production losses and ill thrift, causing significant morbidity and sometimes mortality in young livestock. Control of GIN is currently focused on chemotherapeutic treatment with broad spectrum anthelmintics. The repeated and constant use of these chemicals has put significant selection pressure on GIN resulting in the emergence of resistance to several drug classes [1, 2]. Anthelmintic resistance (AR) is now widespread and threatens the future sustainability of the sheep industry worldwide [3]. In the current climate where AR appears to be inevitable, research is now being focused on slowing the development of resistance on farms.

The importance of conserving refugia of drug-naïve parasites to dilute the eggs of resistant worms surviving treatment was first suggested in the 1980s [4, 5] and has since been highlighted as a key factor determining the rate of development of AR. Several studies have demonstrated the benefits of maintaining an un-selected population of parasites in refugia on conservation of drug efficacy over time and slowing the development of AR [6–8] within species. In addition, a longitudinal study on the impact of different drenching patterns on the performance of grazing lambs [8], demonstrated that growth rates of lambs following targeted treatment regimes aimed at conserving parasites in refugia, were comparable with the growth rates of monthly treated animals. Regular monthly suppressive treatments have been shown to reduce species diversity and worm burden over the course of a single grazing season [9]. This study aimed to investigate the impact of four different ivermectin treatment regimes upon parasite composition when implemented over several consecutive years.

## Methods

### Ethics statement

The study was examined and approved by the Moredun Research Institute Experiments and Ethics Committee and was conducted under the legislation of a UK Home Office Licence (reference PPL 60/03899) in accordance with the Animals (Scientific Procedures) Act of 1986.

### Experimental design

The replicated field trials in this study were conducted in each of six consecutive years (2006–2011) at the Moredun Research Institute farm, Scotland.

Parasite material was collected throughout the 2006 and 2011 seasons of the longitudinal field trial described by Kenyon et al. [8], which investigated the impact of four anthelmintic treatment strategies on drug efficacy and production efficiency in grazing lambs. Briefly, replicated field trials were conducted over six consecutive years (2006–2011); each year, 128–192 lambs were split into eight groups (balanced for weight, sex and faecal egg count) which were randomly allocated to eight one hectare paddocks, replicate groups received one of four ivermectin treatment regimes (Oramec, Merial Animal Health Limited, UK at the manufacturer’s recommended dose rate of 0.2 mg/kg live weight). The eight paddocks were randomly assigned to the four treatment groups at the start of the experiment and treatment strategies were maintained on the same paddocks throughout to compare the impact of different treatment regimes on parasite epidemiology over six years. Implementation of the different treatment strategies began on day 56 of each grazing season (apart from TST in 2006 which began on day 98, due to validation of the Happy Factor [10] model in the first year of the experiment). The molecular species data from trial lambs described in this paper were collected from day 56 until the end of the grazing season (day 154) in the 2006 and 2011 seasons, whilst tracer lambs were used to examine contamination on paddocks at the start and the end of the experiment.

### Treatment strategies

Two groups of lambs received a neo-suppressive treatment (NST) regime in which a whole-group drench was administered every four weeks throughout the grazing season. The second treatment strategy was a targeted selective treatment (TST) regime; individual lambs were drenched based upon their ability to reach individualised target weights. These target weights were generated using the Happy Factor™ decision support model described by Greer et al. [10] which considers environmental factors, food availability and growth rates of individual lambs. The third treatment regime was a strategic prophylactic (SPT) approach where a whole-group drench was administered twice throughout the grazing season; at weaning and 4 to 6 weeks post-weaning. The fourth followed a metaphylactic/therapeutic (MT) treatment strategy; all lambs in both paddocks were drenched only when clinical signs of ill thrift appeared in lambs in either paddock. In 2006, lambs in all treatment groups, except MT, received benzimidazole (Panacur, Intervet at 5 mg/kg live weight) on day 28 to control for *Nematodirus* species infection. In 2011, all lambs received benzimidazole on day 7 and ivermectin on day 28, for the same reasons.

### Sample collection

Two sources of material were used: nematode eggs from faeces collected from trial lambs and worm burdens from tracer lambs. Faecal samples were collected from each lamb at 8 time-points throughout the grazing season to give an indication of species output from the host. Two parasite-naïve ‘tracer’ lambs were grazed on each paddock at the start (May) and another two at the end (September) of every grazing season to indicate the number and species of nematode present on pasture.

#### Nematode egg extraction and DNA extraction

Faecal samples were collected per rectum from each lamb fortnightly and faecal egg counts (FEC) were conducted following the method of Jackson [11] as described in Kenyon et al. [8]. This FEC method provides a sensitivity of up to one egg per gram of faeces (epg). *Nematodirus* eggs were differentiated morphologically from other strongylid species and were not included in the results here as this species does not hatch to L_1_.

Following faecal egg counting, the strongylid eggs present in each 1 g faecal sample were pooled by treatment group, assuming that variability between lambs and paddocks was negligible in accordance with our field experience, to give a pooled representative of the population for each treatment group at each time point. The eggs were washed over a 25 μm sieve with tap water to remove the salt, and incubated in a petri dish at 22 °C for 72 h to allow the eggs to hatch to the first larval stage (L_1_). Hatching was verified microscopically and larvae were fixed in ethanol (final concentration > 70 % EtOH). Ethanol-fixed L_1_ were bathed in 1x phosphate-buffered solution (PBS) (1/100 v/v) for 30 min to rehydrate larvae. Individual L_1_ from samples collected on day 56 onwards were picked and placed into each well of a 96 well plate (Axygen), containing 50 μl lysis buffer (50 mM KCl, 2.5 mM MgCl_2,_ 10 mM Tris (pH 8.3) 0.45 % Nonidet P-40, 0.45 % Tween 20, 0.01 % Gelatine plus 0.1 mg/ml proteinase K) [12]. Forty-two individual larvae, were randomly selected from the pool for each treatment group at each time point. Plates were placed at -20 °C overnight, incubated at 56 °C for 4 h then heated to 95 °C for 15 min to deactivate the proteinase K. DNA was precipitated by adding 100 μl ethanol to each well; plates were kept a -20 °C overnight and centrifuged (4000 *g*, 40 min at 4 °C). Supernatant was removed and plates were air-dried briefly. Extracted DNA was then re-suspended in 50 μl of DNA-free water.

#### Worm burdens from tracer lambs

As described by Kenyon et al. [8], tracer lambs were grazed for 28 days then housed for two weeks before euthanisation. Abomasa, small intestine and large intestine were recovered from each animal. Each organ was opened longitudinally, placed in 5 L of physiological saline (0.85 % sodium chloride w/v) and incubated at 37 °C for 4 h, following which the mucosa was washed into the saline and the organ removed. A 10 % sub-sample was collected from each organ and immediately fixed in 2 % formalin [13].

### Species identification

Species were identified for trial lambs from each of the 42 L_1_ from the pool for each treatment by time point using multiplex PCR or species specific PCR (see below). For each tracer lamb, the worms present in a 2 % organ digest sub-sample were counted and differentiated by developmental stage and sex and approximately 25 males were then cleared using lactophenol (GCC Diagnostics, UK) and species identified in light microscopy morphological examination [13].

#### Multiplex PCR

A published multiplex PCR assay [14] was utilised to identify the five most common nematode species found in this region: *Teladorsagia circumcincta*, *Trichostrongylus vitrinus*, *Trichostrongylus colubriformis*, *Trichostrongylus axei* and *Haemonchus contortus*. PCR reactions (10 μl) were carried out using Platinum Taq (Invitrogen) containing 1.5 mM MgCl_2_, 0.2 mM dNTPs, 0.4 pmol/μl internal transcribed spacer 2 (ITS2) generic primers (ITS2GF, ITS2GR) and 0.3 pmol/μl of each species-specific primer (TECIRV1, TRCORV1, TRVIFD1, TRAXFD2, HACOFD2 as described by Bisset et al. [14]. 1.5 ng of genomic DNA template was added to each reaction. Two PCR-negative (no genomic DNA template), two lysis-negative (worm lysis buffer without L_1_) and five DNA-positive controls were included in each 96 well plate PCR reaction. Positive control DNA was extracted (Qiagen, DNeasy blood and tissue kit, Hilden, Germany) from morphologically identified adult parasites of the species listed above.

A touchdown PCR cycle was conducted as follows: stage one; initial denaturation at 94 °C for 8 min followed by 12 cycles of 94 °C for 10 s, 60–54 °C (decreasing by 0.5 °C per cycle) for 15 s (annealing) and 72 °C for 30 s (extension). Stage two comprised 25 cycles of 94 °C for 10 s, 54 °C for 15 s and 72 °C for 30 s, followed by a final extension phase of 72 °C for 7 min. PCR products were analysed by QIAxcel advanced capillary electrophoresis using the QIAxcel DNA High resolution kit (Qiagen, Hilden, Germany) following the manufacturers’ protocol. Analysis was completed using QIAxcel ScreenGel software (Qiagen, Hilden, Germany).

#### Species-specific PCR

Multiplex PCR included pan-nematode ITS2 primers in addition to the five species-specific primers to highlight the presence of DNA from nematode species not included in the assay. Species-specific PCR reactions were used to screen samples which amplified only a pan ITS2 band for *Chabertia ovina*, *Oesophagostomum venulosum* and *Cooperia curticei* (Table 1)*.* As eggs of *Trichuris ovis* do not hatch until reaching the predilection site, this species was not included in the assays described. All species-specific PCR reactions were performed using NovaTaq Hot Start master mix (Merck, New Jersey, USA) in 10 μl volumes. PCR targeting *C. ovina* and *O. venulosum* contained 5 μl 2× buffer, 3.5 mM MgCl_2_, 1 mM of each primer, 1.5 ng target DNA and ddH_2_0. PCR targeting *C. curticei* contained 5 μl 2× buffer, 1.5 mM MgCl_2_, 0.2 mM of each primer, 1.5 ng target DNA and ddH_2_0. Reactions were incubated at 94 °C for 10 min followed by 35 cycles of 94 °C for 30 s, PCR-specific annealing temperature (Table 1) for 30 s and 72 °C for 30 s, followed by a final extension phase at 72 °C for 10 min. All PCR products were run on a 2 % agarose gel stained with gel red (Bioline, London, UK) and visualised under UV illumination.

**Table 1:** Primer sequences and annealing temperatures (Ta) for species-specific PCR reactions

Species	Primer	Sequence	Ta	Product size	Reference
*Chabertia ovina*	CHO	GATGACCTCGTTGTCACCGTG	55 °C	162 bp	[30]
	NC2	TTAGTTTCTTTTCCTCCGCT			
*Oesophagostomum venulosum*	OEV	TGAAATGAGACAACCGTAGTCG	55 °C	105 bp	[30]
	NC2	TTAGTTTCTTTTCCTCCGCT			
*Cooperia curticei*	CcF	TATACTACAGTGTGGCTAGCG	52 °C	143 bp	[17]
	CcR	TCATACCATTCAGAAATGTTC			

#### Statistical analysis

Multispecies abundance data (six species: *Te. circumcincta*, *Tr. vitrinus*, *Tr. colubriformis*, *H. contortus*, *C. ovina* and *O. venulosum*) were obtained from the 42 L_1_ as described above. The relationships between these pooled abundance profiles and the year (2006, 2011), grazing season (split into two periods: < 105 (mid) and > 105 days (late)) and treatment (NST, MT, SPT and TST) factors were explored by fitting a multivariate regression tree (MRT) [15]. The MRT was based on repeated splitting of the abundance profiles into homogenous groups, according to the categories of the given factors, until an optimal partition was reached which minimized the within-group dissimilarities. These latter were measured by the sum of squared Euclidean distances of the abundance profiles about the group mean abundances (centroids). Equivalently, this maximized the sum of squared Euclidean distances between group centroids. The resulting partition was graphically displayed as a tree-like structure that reveals the relative importance of the factors from top to bottom.

Adult worm burdens from the 4 tracer lambs (2 lambs per paddock) at each stage of grazing for each treatment were modelled using generalised linear models (GLMs) with negative-binomially distributed errors and logarithmic link function. Note that preliminary assessment revealed that fitting a paddock random effect was not needed. We focused on the two most relevant species for which richest data were available (*Te. circumcincta*, *Tr. vitrinus*). The factors year (2006, 2011), season (early, late), treatment (NST, MT, SPT and TST) and interactions between them were entered as fixed effects in initial models for both species. The final GLM for *Te. circumcincta* included year, season, treatment and all their two-way interactions as statistically significant factors. The final GLM for *Tr. vitrinus* included season as the only statistically significant explanatory factor. Model selection was based on likelihood ratio tests and the usual 5 % threshold was used for concluding statistical significance.

All statistical analyses were conducted using the R system for statistical computing v3.2 [16].

## Results

### Species composition of L_1_ from faecal samples

**Fig. 1:**
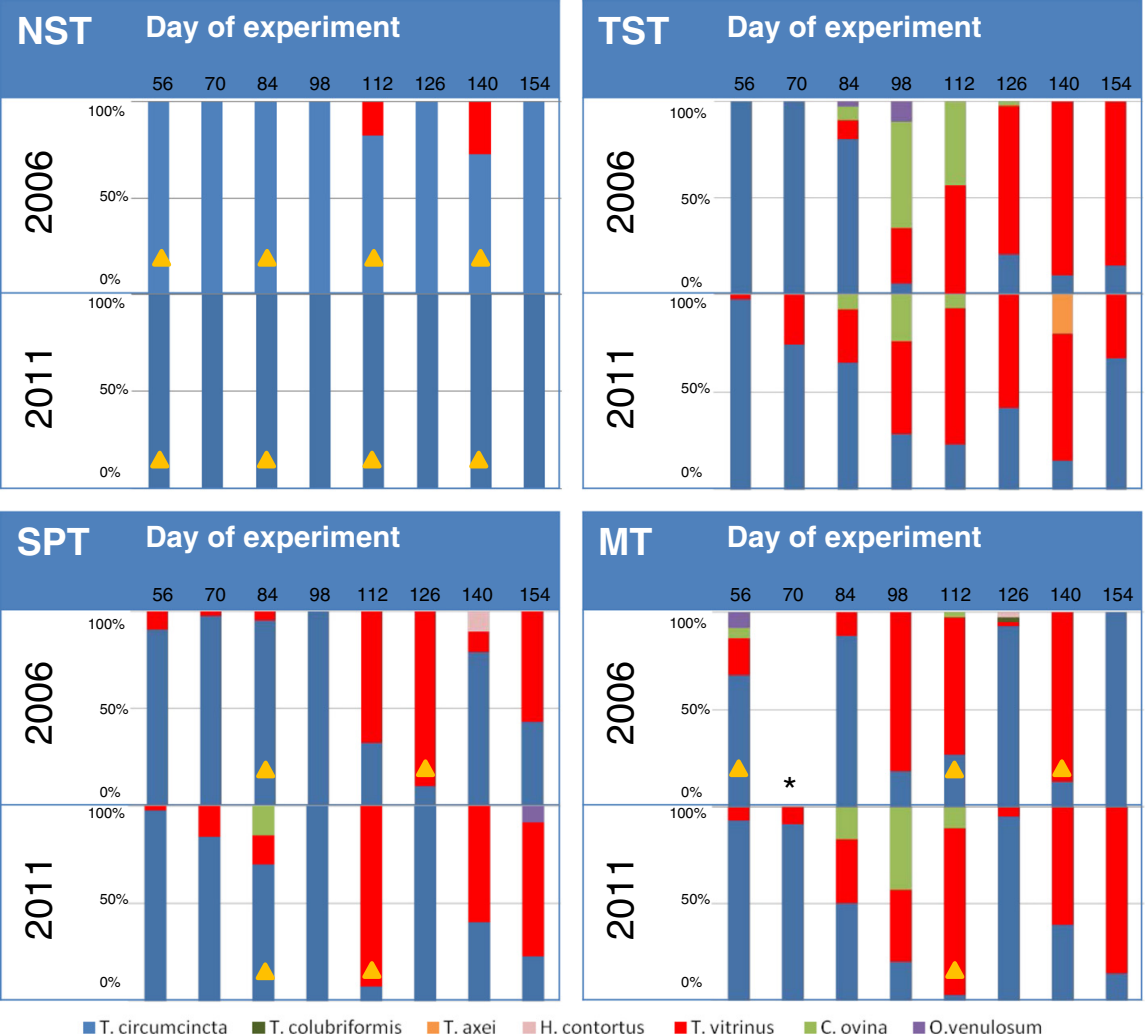
Species composition (%) of first stage larvae (L_1_) hatched from faecal samples collected from trial lambs from Neosupressive (NST), Targeted Selective (TST), Strategic prophylactic (SPT) and Metaphylactic/therapeutic (MT) treatment groups during the grazing seasons in 2006 and 2011. *No sample available for 2006, day 70 MT group. Yellow triangle, Ivermectin treatments administered to all lambs in the treatment group. In both years, varying numbers of lambs were drenched in the TST group, according to their ability to meet individualised predicted weight gain targets, see Table 3

Figure 1 shows the percentage of each species identified from each of the eight sampling periods in 2006 and 2011 from trial lambs in each of the four treatment groups.

In 2006, there was a range of strongylid species present in all treatment groups during the season. The predominant species in all groups at day 56 of the experiment was *Te. circumcincta*, which accounted for 100, 100, 90.4 and 67.6 % of L_1_ recovered from faecal samples in the NST, TST, SPT and MT group, respectively. Later in the season, a greater variety of nematode species were identified. *Trichostrongylus vitrinus* was the predominant species from day 98 in the MT group and day 112 in the TST and SPT groups, comprising 80, 56 and 67 % of strongylid larvae, respectively. Other species were found to a lesser extent and varied between groups; for example, *C. ovina* was present in the TST group in mid-season, was not observed in the SPT group and was rare in the MT group. *Haemonchus contortus* was identified at two sampling points, day 140 in the SPT group and day 126 in the MT group. In comparison, *Te. circumcincta* continued to dominate in the NST group, with the only other species identified being *Tr. vitrinus* observed on day 112 and 140 of the experiment, with a relative abundance of 17.5 and 27.5 % respectively.

In 2011, the species composition of samples collected from the TST, SPT and MT groups were broadly similar to those observed in 2006, except that *Tr. vitrinus* appeared earlier in the season, from day 56 onwards. *Chabertia ovina* and *Oesophagostomum venulosum* were observed in the SPT and TST groups, but were absent from the MT group. In contrast, only *Te. circumcincta* was identified from the L_1_ collected from faecal samples of NST group lambs. *Trichostrongylus axei* was not identified in any group at any point.

**Fig. 2:**
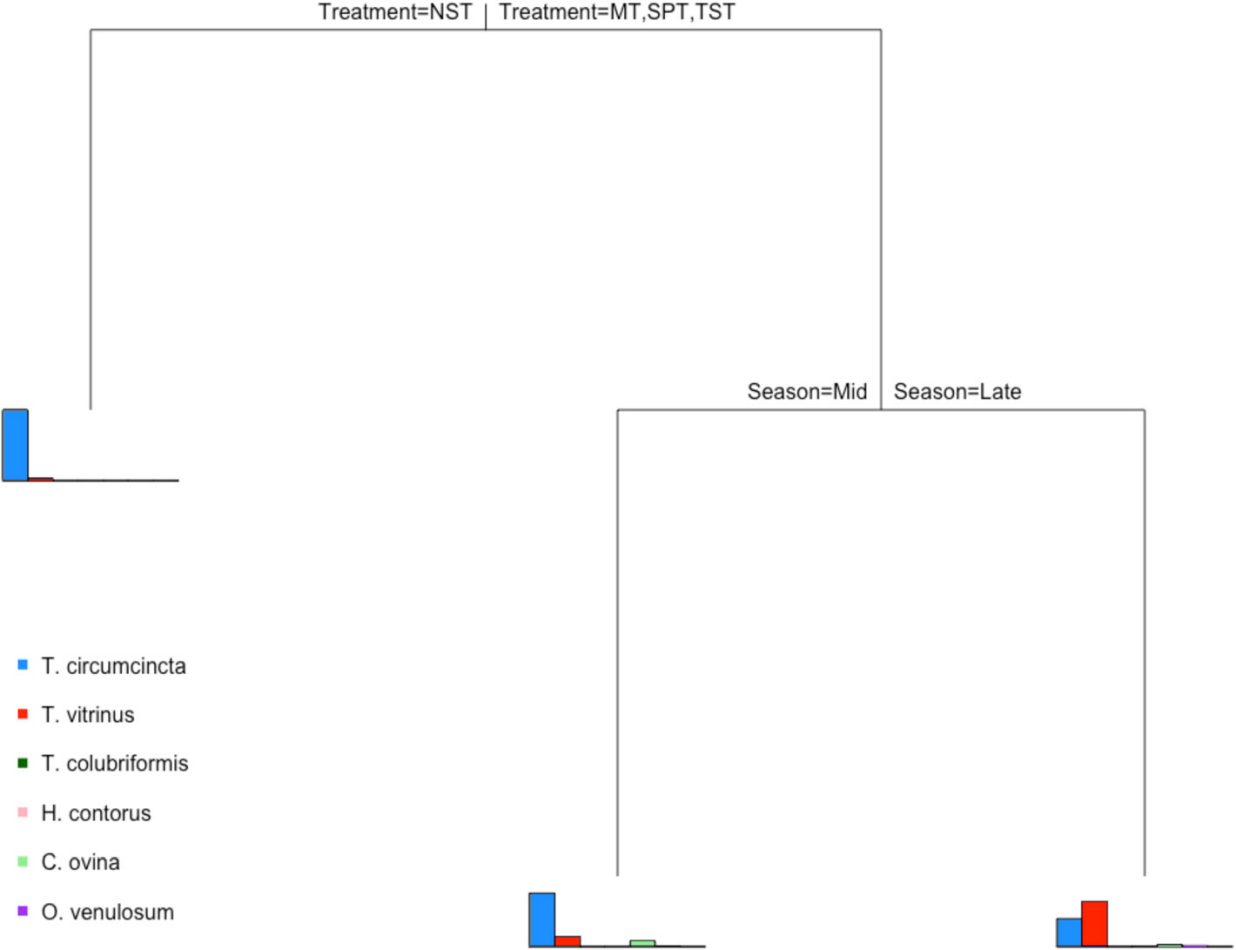
Multivariate regression tree showing the hierarchical structure of homogeneous groups of species abundance profiles of first stage larvae (L_1_) hatched from faecal samples collected from trial lambs when comparing by years, seasons and treatment groups. The bar plots at the terminal nodes show the mean species composition of each of the three groups differentiated according to treatment and season

Figure 2 displays the tree-like grouping structure resulting from the MRT analysis. Three homogenous groups were distinguished with the splits based only on treatment and season. The barplots at the terminal nodes represent the mean species distributions per group. The main distinction was due to treatment, placed on the top node of the tree, with the strong dominance of *Te. circumcincta* (mean prevalence of 97.1 %) and the minimal presence of any other species (only *Tr. vitrinus*, mean prevalence of 2.9 %) in the abundance profiles resulting from the NST treatment determining the first split (19 % reduction in within-group dissimilarity). The other three treatments (MT, SPT and TST) formed a separate homogeneous group of abundance profiles, which was only further split according to season (30.5 % reduction in within-group dissimilarity). In the mid-season, *Te. circumcincta* was the dominant species, accounting on average for 77.2 % of L_1_ passed out onto pasture, whereas *Tr. vitrinus*, *C. ovina* and *O. venulosum* accounted for 14.2 %, 7.6 % and 0.9 % of L_1_, respectively. Finally, in the group at the rightmost terminal node, which corresponds to cases under treatment MT, SPT or TST and late season, a more varied range of species was found. The mean relative abundances of the observed species were 36.9 % *Te. circumcincta*, 58.8 % *Tr. vitrinus*, 0.1 % *Tr. colubriformis*, 0.3 % *H. contortus*, 2.7 % *C. ovina* and 1.2 % *O. venulosum*.

### Species composition of adult worms recovered from tracer lambs

Table 2 shows the mean totals and percentage species composition of adult worms identified from the tracer lambs which had grazed the respective paddocks. The most prevalent nematode species in the TST, SPT and MT groups was *Te. circumcincta*, accounting for 47.9 %, 73.1 % and 81.1 % of the strongylid nematodes recovered, respectively. *Teladorsagia circumcincta* comprised 25 % of worms recovered from the NST group early 2006 tracer lambs; the most prevalent nematode species in this group were *Tr. vitrinus* and *T. ovis*, comprising 28.1 and 26.4 % of worms, respectively. Species composition in late 2006 remained similar in all groups with the exception of NST in which *Tr. vitrinus* was reduced in relative terms, comprising 15.2 % of the total worm burden compared to 50.6, 44.3 and 45.8 % in the TST, SPT and MT groups, respectively. Comparison of total worm burdens from the late season tracer lambs in 2006 and 2011 indicated an increase in pasture contamination over the course of the study in the NST and MT groups and a reduction in the TST and SPT groups.

**Table 2:** Mean (± SEM) total worm burden and mean percentage prevalence of strongylid species present over four tracer lambs in each of the 4 treatment groups and each year and season of the study. ‘-‘means that the species was not present

Group	Year	Season	Total worm burden	% *Te. circ*	% *Tr. axei*	% *Tr. vit*	% *Tr. col*	% *O. ven*	% *C. ovina*	% *T. ovis*
NST	2006	Early	1488 (±597)	25.0	–	28.1	–	8.8	11.7	26.4
		Late	12,184 (±4473)	79.9	4.1	15.2	–	–	–	0.8
	2011	Early	2594 (±774)	94.8	1.7	2.5	–	–	1.0	–
		Late	23,954 (±3942)	94.1	2.2	3.8	–	–	–	–
TST	2006	Early	2537 (±1206)	47.9	–	7.7	–	9.8	31.1	3.5
		Late	36,317 (±20,307)	48.6	0.8	50.6	–	–	–	–
	2011	Early	5929 (±2277)	52.2	–	47.8	–	–	–	–
		Late	14,586 (±3436)	63.9	–	36.1	–	–	–	–
SPT	2006	Early	1548 (±1151)	73.1	–	7.3	–	–	5.9	13.7
		Late	41,182 (±20,304)	54.8	–	44.3	–	0.9	–	–
	2011	Early	2770 (±955)	70.2	–	29.8	–	–	–	–
		Late	27,740 (±4259)	73.3	0.7	25.9	–	–	–	0.1
MT	2006	Early	9016 (±3408)	81.1	–	7.1	0.3	5.7	4.4	1.4
		Late	19,181 (±7313)	53.6	–	45.8	–	–	–	0.5
	2011	Early	9694 (±2393)	79.8	–	20.2	–	–	–	–
		Late	37,834 (±7184)	40.4	0.4	58.9	–	0.2	–	0.1

Differences between the treatment groups were evident in worm burdens collected from tracer lambs grazing on the paddocks at the beginning of the grazing season in 2011. The most predominant GIN species was still *Te. circumcincta*, comprising 94.8, 52.2, 70.2 and 79.8 % of total worm burden in NST, TST, SPT and MT groups, respectively. Species diversity was similar to that found in early 2006 for TST, SPT and MT groups; however, a reduction in the relative abundance of the large intestinal species (*O. venulosum* and *C. ovina*) was observed in all groups, from being present at low prevalences in early season 2006 (range 4.3–31.1 for both species over the 3 groups) to 1 % or less in early season 2011. Worm burdens recovered from tracer lambs grazed on NST pastures in 2011 showed that the nematode population on these pastures was more diverse than expected from the data obtained from the egg output (Fig. 1). Although *Te. circumcincta* was by far the most prevalent species, accounting for over 94 % of the worms identified in the NST group, *Tr. vitrinus*, *Tr. axei* and *C. ovina* were also identified at low relative abundance.

Focusing on *Te. circumcincta*, statistically significant interaction effects of treatment group with year (*χ*^*2*^ = 8.95, *df* = 3, *P* = 0.030) and season (*χ*^*2*^ = 14.52, *df* = 3, *P* = 0.002) on the mean abundance of *Te. circumcincta* were found. Statistical comparison in 2006 worm burdens revealed no statistically significant overall differences between treatment groups regardless of the season (*χ*^*2*^ = 5.22, *df* = 3, *P* = 0.157). However, overall *Te. circumcincta* burdens recovered from tracer lambs grazed on the pasture at the end of 2006 were significantly higher in the late season (*χ*^*2*^ = 45.96, *df* = 1, *P* < 0.001). In 2011, a significant interaction between treatment group and season was found (*χ*^*2*^ = 19.01, *df* = 3, *P* < 0.001). In early 2011, statistically significant differences between group means were found (*χ*^*2*^ = 14.23, *df* = 3, *P* = 0.003). The overall relative abundance of *Te. circumcincta* in the NST group increased from 2006, reaching a mean level in comparison with the other groups in 2011 (*χ*^*2*^ = 8.95, *df* = 3, *P* = 0.030); however *Te. circumcincta* populations remained stable in the TST, SPT and MT groups between years.

With regard to *Tr. vitrinus*, only a statistically significant effect of season was found (*χ*^*2*^ = 17.36, *df* = 1, *P* < 0.001), with a higher predicted mean overall worm burden, as expected, at late season: 7800 [CI: 4074.52–14931.84] versus 860.05 [CI: 449.20–1646.66]).

### Effect of anthelmintic drenching

The timings of whole-group anthelmintic drenching are shown for the NST, SPT and MT groups in Fig. 1. The proportion of the TST group treated at each sampling point is shown in Table 3. In 2006, only *Te. circumcincta* was observed from the cultured L_1_ post ivermectin administration in the NST group. In the SPT and MT groups, anthelmintic use changed the species composition so that *Te. circumcincta* predominated, however other species continued to be present at a low relative abundance at some time points. The species composition in the TST group did not show a defined change in species composition (Fig. 1), even when 69 % of the group was drenched (see Table 3).

**Table 3:** The percentage of lambs in the TST group drenched at each sampling point in 2006 and 2011. TST treatment was implemented from days 98 and 56 onwards in 2006 and 2011, respectively

Day	56	70	84	98	112	127	140	154
2006	–	–	–	16.7	68.8	27.1	68.8	20.8
2011	17.9	33.3	30.8	43.6	51.3	30.8	56.4	41.0

In 2011, only *Te. circumcincta* was identified pre- and post-drenching from the NST group. Drenching in the SPT group, resulted in only *Te. circumcincta* being identified, and in the MT group the species dynamics switched from predomination of *Tr. vitrinus* to predomination of *Te. circumcincta*. Again, no obvious change in species composition was observed in the TST group, even after drenching over 50 % of the group on days 112 and 140.

## Discussion

The aim of this study was to determine the impact of anthelmintic treatment strategy on ovine nematode species composition. Samples were collected to allow the examination of the strongylid nematode species passed out from the host (L_1_ cultured from faeces) and of those present on pasture that were available to infect the hosts (adult worms from worm-naïve tracer lambs) in an attempt to determine the impact of different ivermectin drenching strategies on species composition. Although the use of L_1_, instead of eggs, could potentially have affected the species composition due to some species not developing whilst in culture, the eggs were cultured for 72 h which exceeds the 36–48 h stated to obtain maximum L_1_ hatching rate used by Burgess et al. [17], so we expect that few viable eggs would remain unhatched. Bias could also have been introduced by the selection of 42 larvae for PCR analysis for species identification. This number of larvae was selected to balance sample size with the time and labour costs of picking individual L_1_ for analysis, which was a requirement of the multiplex PCR methodology [14] used. New species identification methodologies which allow for species identification from pooled samples [18–20] could increase the sample size whilst reducing time required for preparation and analysis.

Species profiles obtained from the tracer lamb worm burdens and L_1_ from faecal samples were different. However, this is to be expected due to the differences in the samples from which they originate. The adult worm burdens represent the parasite population on pasture, as tracer lambs are parasite naïve, infection with all species present can occur equally. The L_1_ were obtained from faecal samples collected from trial lambs which have grazed the pasture for the entire season. The L_1_ species profile represents parasites being passed out onto pasture by the animals and is therefore impacted by several factors including developing immunity in the growing lamb and previous anthelmintic drenches administered.

The pattern of GIN species observed in Scotland is typically *Nematodirus* spp. in early spring followed by *Teladorsagia* spp. then *Trichostrongylus* spp. in late summer [21]. This pattern was replicated in our study with the predominant species being *Te. circumcincta* in the early to mid-season and *Tr. vitrinus* becoming more prevalent towards the end of the grazing season in the TST, SPT and MT groups, consistent with previous studies [17, 22]. Seasonal shifts in species prevalence result from a combination of factors including the development of acquired immunity in lambs [23, 24] and environmental shifts in temperature and rainfall impacting the hatching of nematode eggs on pasture [25]. Comparable species profiles were obtained from all treatment groups in the 2006 early season, showing that the populations were similar at the start of the experiment in both species composition and contamination levels. In contrast, there was a marked difference in the prevalence of nematode species identified both from the host and from pasture between the NST and other treatment groups throughout 2011. As all variables except anthelmintic use were similar for all pastures (environmental and stocking conditions etc.), the reasons for this change must be the anthelmintic strategy applied.

There appear to be clear differences in the species composition in egg output of the trial lambs between the anthelmintic treatment groups in 2006, with the NST showing only *Te. circumcincta* and *Tr. vitrinus* compared to the TST, SPT and MT groups in which a greater range of species, including the anthelmintic susceptible large intestinal nematodes, was identified. This restriction of species was continued in 2011 in the NST group, with only *Te. circumcincta* being identified, whereas the other groups maintained a greater number of species. The removal of nematode diversity from the NST group is likely due, in part, to the frequency of anthelmintic treatments administered over each grazing season which is in agreement with Leathwick et al. [9], which showed reduced species diversity in a single grazing season. Fewer anthelmintic drenches were administered to the TST, SPT and MT groups within each grazing season thus, a significantly lower selection pressure was placed upon the parasites in these groups and as a result, species diversity was largely maintained. Co-infections with multiple nematode species, the normal state in sheep, has been found to alter the establishment of various nematode species when compared with single species infections e.g. *Te. circumcincta* and *Tr. vitrinus* [26] and thus within-host competition may help to regulate the effects of infection [27]. Monospecific infection has been associated with production limiting disease [28], therefore the maintenance of multi-species infection could be important for effective control.

Ivermectin treatment in all groups resulted in an increase in the proportion of *Te. circumcincta* in L_1_ samples in the two weeks post treatment, the predominant species associated with ivermectin resistance in Scotland [29]. Diversity appears to be restored after a four-week period, in all groups apart from NST, presumably due to re-infection from suprapopulation which was shown by worm burdens to retain species diversity. Only *Te. circumcincta* was identified in post treatment egg output samples in NST, probably as the other ivermectin-susceptible species were cleared by anthelmintic treatments administered early in the season, prior to implementation of the different treatment strategies on day 56. The potential reasons for the rapid (within single season) change in species composition in the NST group was likely due to the frequency of drenching which removed the susceptible nematode species from the host. Furthermore, as these species would be removed early in the season, their ability to re-infect pasture would be reduced, compared to the drug-resistant *Te. circumcincta*. Indeed, the proportion of *Te. circumcincta* on pasture increased in the NST group during the course of the study, with over 90 % of the worm population on pasture being *Te. circumcincta* throughout 2011. The rapid impact of monthly drenching on the GIN population within a single grazing season is in agreement with previously published observations [9].

The TST, SPT and MT treatment regimes were designed to maximise refugia by targeting treatment to individual animals that would benefit from drenching or timepoints where the majority of animals would benefit. The presence of a susceptible parasite population in refugia has been highlighted as a key factor in determining the rate of development of AR [4, 5]. Drug-naïve, un-selected parasites in refugia dilute resistant survivors of treatment and thus delay the emergence of AR. IVM-susceptible species such as *C. ovina* and *Tr. vitrinus* were identified in both egg output and worm burden samples collected from the TST, SPT and MT groups in 2011: the conservation of these species over time, especially in the whole-flock treatment groups, confirms that these parasites were surviving in refugia. The impact of whole-group treatments can be seen in the reduction in species diversity detected two weeks post-treatment in the SPT and MT groups (days 84 and 112 respectively, 2006). The species surviving treatment was predominantly *Te. circumcincta*; however, species diversity was restored four weeks post-treatment through re-infection from the rich population of parasites in refugia. Species diversity was also restored in the NST group following drenches in 2006; however, in 2011, only *Te. circumcincta* was observed in L_1_ cultures throughout the grazing season. *Trichostrongylus axei*, *Tr. vitrinus* and *C. ovina* persisted at low levels on pasture in the NST group throughout the experiment; they were identified from NST tracer lambs in 2011. The lack of these species in the L_1_ cultures is likely due to the frequency of anthelmintic treatments administered preventing successful re-infection of the trial lambs. Given the sampling method used to examine the L_1_ species composition, it is possible that additional species were present in the NST group trial lambs in very low numbers however, the data illustrates that *Te. circumcincta* is the prominent species being passed out onto pasture throughout the season, resulting in a greater expansion of this population compared to other species. This is probably due to the fact that drug resistant nematodes that are unaffected by the drench will continue to lay eggs with little or no interruption. However for any drug susceptible nematode species, reinfection will have to occur and the resulting approximately 21 day pre-patent period means that egg output from these species will be delayed in comparison to the drug resistant nematodes. For example, in the NST group it may mean that *Te. circumcincta* is able to have continuous egg output throughout the whole experimental period, whereas egg output from drug susceptible species will only occur for one of the four weeks between drenches. The relative abundance of *Tr. vitrinus* in the NST group in 2006 shows a reduction compared to the other three treatment groups. Adult worm burdens collected at the end of the 2011 grazing season confirm that the *Tr. vitrinus* population is markedly reduced in the NST group, therefore suggesting that the refugia of supraparasites has been reduced in this treatment group over the six years of the experiment.

On average 26 % of lambs were treated at each timepoint in the TST group [8] thus, refugia was maintained both on pasture and within un-treated animals. The part-flock treatment method used in this group would be expected to provide a greater refugia than the whole-flock treatment methods as, in theory, fewer eggs from drench-surviving parasites will be passed out onto pasture and therefore eggs from nematodes surviving treatment would be further diluted. Despite leaving a proportion of the animals untreated at each time point, pasture contamination in the TST group was lower than that of the other treatment groups at the end of the experiment. Species composition of egg output from TST trial lambs appears more uniform however, the overall nematode species composition in 2011 was similar to that of the SPT and MT groups. It can therefore be concluded that refugia can be successfully maintained using a variety of different management approaches.

Kenyon et al. [8] reported a reduction in drug efficacy in all groups by 2010, where efficacies were 62, 86, 86 and 83 % for the NST TST, SPT and MT groups, respectively. The predominance of *Te. circumcincta* post-treatment strongly suggests that IVM resistant *Te. circumcincta* were present in all treatment groups. Other species present in the 2011 SPT and MT pre-treatment samples appear to be successfully removed by each treatment. Larvae of *Tr. vitrinus* were identified in small numbers in some SPT and MT post-treatment samples; however, the proportion of larvae of this species is greatly reduced post-treatment and often not detected at all, suggesting that *Tr. vitrinus* has not developed resistance to IVM over the period of study. IVM efficacy for each species cannot be reliably calculated due to the sampling method used for species identification. A total of 42 individual larvae were analysed from each treatment at each time point, and whilst this small sub-sample gives an indication of the species composition, it cannot provide a reliable quantification of the prevalence of the individual species. The overall drug efficacy in the TST, SPT and MT groups remained higher than that of the NST group throughout the study [8], likely due to the presence of susceptible parasite species in refugia.

## Conclusions

Anthelmintic resistance is thought to be inevitable in the current parasitic climate due to the repeated and intensive use of chemotherapeutics. This study builds upon the findings of Kenyon et al. [8], highlighting the impact of different treatment strategies on refugia of GIN over time, using molecular species identification techniques. Results suggest that frequent whole-group drenches reduce the diversity and number of GIN surviving in refugia, associated with an increased rate of development of AR. The results also show that maintenance of a rich refugia of susceptible parasites can be achieved both by part-flock, targeted selective treatments or strategically-timed whole-group treatments. One of the key factors in reducing the rate of development of AR is the presence of a refugia of drug susceptible parasites. This study has illustrated that this can be successfully achieved through a variety of management strategies.

## Abbreviations

AR: anthelmintic resistance
EPG: eggs per gram
EtOH: ethanol
FEC: faecal egg count
GIN: gastrointestinal nematode
ITS2: internal transcribed spacer 2
IVM: ivermectin
L_1_: first developmental stage larvae
MT: study group receiving treatment upon observation of clinical signs of ill thrift
NST: study group receiving neo-suppressive monthly treatment
PBS: phosphate-buffered solution
PCR: polymerase chain reaction
SEM: standard error of the mean
SPT: study group receiving strategic prophylactic treatment
Ta: annealing temperature
TST: study group receiving targeted selective treatment
